# FLIP regulation of HO-1 and TNF signalling in human acute myeloid leukemia provides a unique secondary anti-apoptotic mechanism

**DOI:** 10.18632/oncotarget.168

**Published:** 2010-09-13

**Authors:** Stuart A Rushworth, Lyubov Zaitseva, Susana Langa, Kristian M Bowles, David J MacEwan

**Affiliations:** ^1^School of Pharmacy, University of East Anglia, Norwich NR4 7TJ, United Kingdom; ^2^Department of Haematology, Norfolk and Norwich University Hospitals NHS Trust, Colney Lane, Norwich, NR4 7UY, United Kingdom

**Keywords:** leukemia, apoptosis, transcription factor, capsases, modulatory protein

## Abstract

Acute myeloid leukemia (AML) comprises a heterogeneous group of clonal disorders of hematopoietic progenitors. We previously showed that heme oxygenase-1 (HO- 1/Hsp32) underlies resistance of AML to TNF-induced apoptosis. Here we show for the first time that the modulatory protein, FLICE-inhibitory protein (FLIP) indirectly regulates induction of HO-1 in response to TNF in human AML blasts, but not noncancerous control cells. In AML cells, TNF-induced FLIP expression was an NF-κB-dependent event, and silencing of FLIP isoforms (FLIP_L_, FLIP_S_ and FLIP_R_) induced pro-apoptotic responses to TNF, with FLIP_L_ knock-down providing the greatest apoptotic switch. However, FLIP_L_ knock-down consequently increased expression of HO-1; a response that occurred in AML (but not non-cancerous) cells to protect a proportion of them from apoptotic death. Our results show that increases in HO-1 induced an apoptotic-resistant form in AML cells in the absence of FLIP_L_. This is the first time that FLIP_L_ has been shown to regulate the expression of HO-1. These data reveal unique regulatory networks in cancerous AML cells whereby FLIP regulation of HO-1 provides AML cells with secondary anti-apoptotic protection against extrinsic factors (eg TNF/chemotherapies) that try to switch on death signals in these highly death-resistant cells. Future AML therapies should target these mechanisms.

## INTRODUCTION

Acute myeloid leukemia (AML) is a malignancy of the myeloid progenitors, characterized by the rapid growth of abnormal myeloid cells that accumulate in the bone marrow and blood. AML is the most common acute leukemia affecting adults, and its incidence increases with age with 75% of patients diagnosed after the age of 60 [1]. Furthermore, with current intensive therapeutic strategies generally limited to a minority of younger, fitter patients, there is a significant unmet need for better tolerated anti-AML therapy [2]. Therefore understanding the processes that protect these complicated cells from undergoing apoptosis in response to endogenous signals is an essential step to developing new improved anti-AML therapy.

Apoptosis is an essential part of mechanisms that maintain normal cellular homeostasis. Deregulation of the apoptosis machinery and evasion of apoptosis is a mechanism of most human malignancies, including AML [3,4]. Most systemic anti-cancer therapies (SACT) act by the induction of apoptosis, therefore, evasion of apoptosis is mainly responsible for the insufficiency of current therapies. It is well documented that malignant cells can die of apoptosis primarily through the extrinsic death receptor-induced pathway [5], the activation of which involves ligation of a death ligand with trimerization of death receptors, leading to the formation of the death-inducing signaling complex (DISC) followed by the activating cleavage of caspase-8 in the DISC [6]. Apoptosis mediated via DISC is regulated by cellular Fas-associated protein with death domain-like IL-1β-converting enzyme inhibitory protein (FLIP)[7].

FLIP mediated regulation of the DISC is through the modulation of activation of procaspase-8 and thereby prevents induction of apoptosis mediated by death receptors [8]. Inducible control of FLIP expression is thought to be controlled by the transcription factor nuclear factor-κB (NF-κB) however we have shown that other transcription binding sites do exist in the FLIP promoter [9]. The human FLIP gene, with an estimated size of ~48000 base pairs, consists of at least 14 exons. It has been described that at least 11 different isoforms of cellular FLIP are transcribed at the mRNA level, generating different splice variants with only three of these splice variants having been detectable as protein (FLIP_L_, FLIP_S_ and FLIP_R_)[9]. The FLIP isoforms have been shown to have conflicting roles in the regulation of apoptosis at the DISC. While FLIP_S_ and FLIP_R_ have been shown to have a clear anti-apoptotic function, the role of FLIP_L_ in regulating apoptosis has been found to be quite controversial [10,11]. Most reports describe FLIP_L_ as an anti-apoptotic molecule that blocks caspase activation, whereas, others suggest that FLIP_L_ catalyzes caspase-8 processing at the DISC and therefore has a pro-apoptotic function.

We have previously shown that in the human AML cell line THP-1, FLIP_L_ is induced in a NF-κB-dependent response [3]. In the same study we showed that FLIP_L_ had only a limited role in regulating apoptosis in AML cells in response to TNF-mediated death responses. Here we demonstrate a novel pathway involving FLIP_L_ negatively regulating the expression of heme oxygenase-1 (HO-1), whose role in chemoresistance, both in AML and other cancers, is well established.

## RESULTS

### FLIP isoform expression in human AML in response to TNF

**Figure 1:**
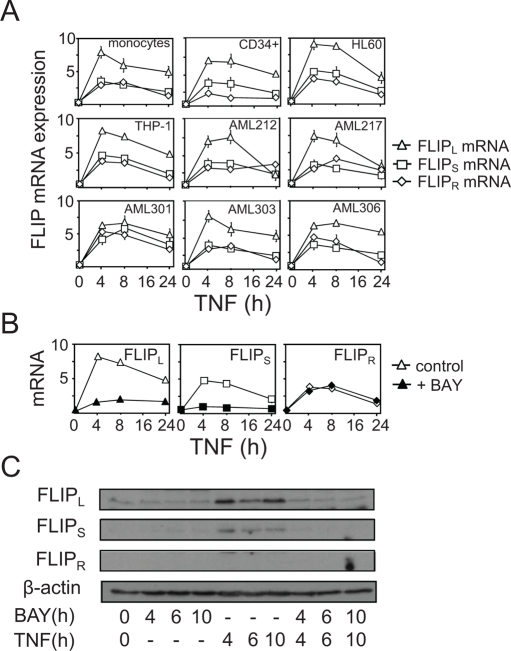
Differential regulation of FLIP isoform expression by TNF in human AML (A) AML samples, AML cell lines and non-malignant control cells were treated with TNF (10 ng/ml) for up to 24 h. Total RNA was extracted, reverse transcribed and relative expression of FLIP_L_ (triangles), FLIP_S_ (squares) and FLIP_R_ (diamonds) mRNA expression was measured by real-time PCR. Data represent the means ± SEM, from three separate experiments. (B) THP-1 cells were treated without (open symbols) or with BAY 11-7082 (10 μM, closed symbols) for 24 h prior to treatment with 10 ng/ml TNF for the indicated times. Total RNA was extracted, reverse transcribed and relative expression of FLIP_L_, FLIP_S_ and FLIP_R_ mRNA expression was measured by real-time PCR. (C) Whole cell Western blot analyses performed for FLIP_L_, FLIP_S_ and FLIP_R_ on THP-1 cells in response to treatments with BAY 11-7082 (10 μM) 1 h prior to 10 ng/ml TNF for the indicated times. β-actin levels were used as loading controls to compare equal loading.

Previously we have shown that the FLIP isoform, FLIP_L_, is induced in AML cell line THP-1 and HL60, in response to TNF in a NF-κB-dependent mechanism [3]. Others have shown that cFLIP can protect cells from TNF induced apoptosis [12]. Moreover the function of the three recognised FLIP isoforms (FLIP_L_ FLIP_S_ and FLIP_R_) has not been wholly established in human AML. Therefore, we wanted to fully elucidate the induction of FLIP isoforms (FLIP_L_ FLIP_S_ and FLIP_R_) in response to TNF in human AML patient samples, AML cell lines and non-malignant control cells. Cells were treated with TNF for various time points Figure 1A shows the mRNA induction of FLIP isoforms in response to TNF in human AML cells. This shows that FLIP_L_, FLIP_S_ and FLIP_R_ are all induced by TNF in a time dependent fashion, with all FLIP isoforms mRNAs being significantly raised from at least 4 h treatment with TNF. FLIP_L_ has the greatest induction in all cells tested suggesting that this protein may have the most influence on apoptotic pathways. Figure 1B confirms that the induction of FLIP_L_ and FLIP_S_, but not FLIP_R_ by TNF is a NF-κB-dependent response. The slight but significant mRNA induction of FLIP_R_ by TNF is not controlled by NF-κB. Figure 1C shows the protein induction of these proteins in THP-1 cells confirming that NF-κB regulates this response. One interesting observation is that we could not detect the protein for FLIP_R_, thus suggesting that mRNA is not processed into protein or the protein level of FLIP_R_ is lower than the detectable concentration for this assay.

### siRNA silencing of FLIP isoforms induced differential apoptotic responses to TNF

**Figure 2:**
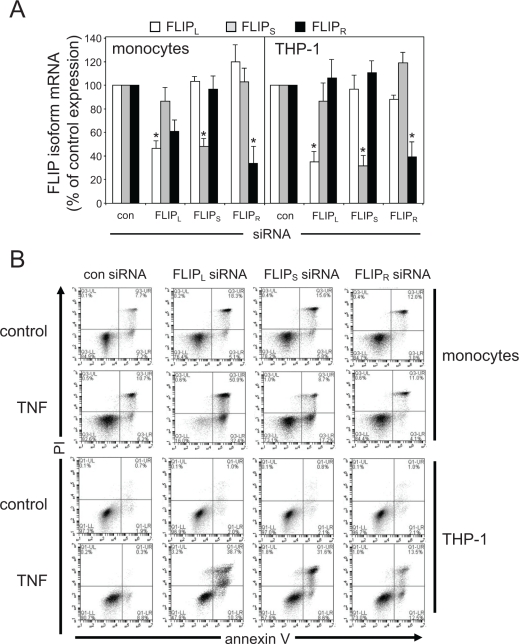
Differential effects of silencing FLIP isoforms on TNF apoptotic responses (A) Human monocytes and THP-1 cells were transfected with 30 nM of either control siRNA or FLIP_L_, FLIP_S_ or FLIP_R_-specific siRNA for 24 h. Total RNA was extracted, reverse transcribed and relative mRNA expression of the indicated FLIP_L_, FLIP_S_ or FLIP_R_ isoform was measured by real-time PCR. Data represent means ± SEM, from three independent experiments. (B) Human monocytes and THP-1 cells were transfected with 30 nM of either control siRNA or FLIP_L_, FLIP_S_ or FLIP_R_-specific siRNA for 24 h before treatment with 10 ng/ml TNF for 24 h. Apoptosis was measured using annexin V and propidium iodide (PI) staining followed by flow cytometry analysis.

To understand the significance of TNF-induced FLIP_L_, FLIP_S_ and FLIP_R_ expression in human AML we used siRNA to target knockdown of these modulators. Figure 2A shows that we can effectively and specifically knockdown mRNA levels of FLIP_L_, FLIP_S_ and FLIP_R_ in human AML cells. When we knocked down FLIP_L_, FLIP_S_ and FLIP_R_ expression in AML cell line THP-1 and human monocytes (Figure 2B) we showed there to no difference in apoptotic response between control knockdown and FLIP_L_ or FLIP_S_ or FLIP_R_ knockdown in THP-1 cells, however we did see a significant increase in monocyte apoptosis in FLIP_L_-targeted cells. When we activated transfected cells using TNF, we found that cells targeted with FLIP_L_ knockdown, but not FLIP_S_ and FLIP_R_, were susceptible to apoptosis. Nevertheless, in this assay, the cells treated with FLIP_L_ knockdown in combination with TNF showed only 44% apoptosis, thus suggesting that some other mechanism is controlling apoptotic responses in the remaining 56% of live cells. However, these findings do suggest that FLIP_L_ but not FLIP_S_ or FLIP_R_ can partially regulate TNF-induced apoptosis in human AML cells

FLIP_L_-targeted knockdown induced HO-1 expression.

**Figure 3:**
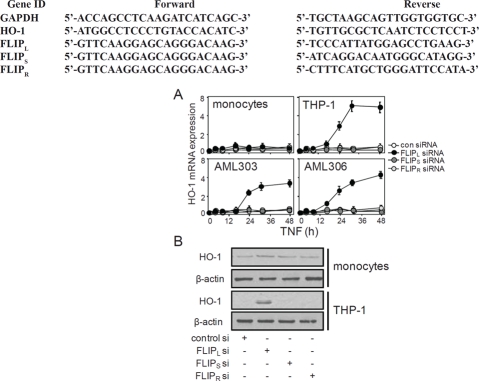
The effect on HO-1 expression of silencing FLIP_L_ isoform, alone and in response to TNF (A) AML samples, AML cell lines and non-malignant control cells were transfected with 30 nM of either control siRNA or FLIP_L_, FLIP_S_ or FLIP_R_-specific siRNA for 48 h, then HO-1 RNA was analysed using real-time PCR. Data represent means ± SEM, from three separate experiments. (B) Human monocytes and THP-1 cells were transfected with 30 nM of FLIP_L_-specific siRNA for 30 h, followed by Western blot analysis for HO-1 protein expression. β-actin levels were also used to compare equal protein loading among samples.

Since we have shown that constitutive expression of HO-1 is regulated by NF-κB in human AML and that FLIP is known to play a role in controlling NF-κB induction we wanted to determine if FLIP knockdown could induce HO-1 expression [13,14]. We used siRNA to knockdown the FLIP isoform expression in two AML samples (AML303 and AML306), as well as in the AML cell line THP-1 and non-malignant control cells. Figure 3A shows that when we knockdown FLIP_L_ protein, we induce expression of cytoprotective genes HO-1 in AML303, AML306 and THP-1, but not control cells. Figure 3B confirms these findings at the protein level. Taken together these data suggest that under normal basal conditions in AML, FLIP_L_ normally regulates the expression of HO-1 in cancerous cells only.

Enhanced apoptotic responses to TNF by silencing FLIP_L_ in combination with HO-1.

**Figure 4:**
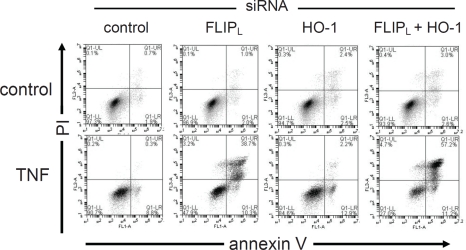
Silencing FLIP_L_ in combination with HO-1 allows enhanced apoptotic responses of TNF (A) THP-1 cells were transfected with 30 nM of either control siRNA or FLIP_L_-specific siRNA in combination with HO-1-specific siRNA for 24 h and then treated with 10 ng/ml TNF for 24 h. Cellular apoptosis was measured by annexin V and propidium iodide staining followed by flow cytometry analysis.

Finally we wanted to determine if silencing FLIP_L_ and HO-1 in combination could induce further apoptosis in AML cells in response to TNF. When we knocked down FLIP_L_ and HO-1 in AML cell line THP-1 (Figure 4) we showed that TNF induced a significantly greater apoptotic response in these AML cells when compared to cells with separate knockdown of either FLIP_L_ or HO-1 alone. Preventing the function of both FLIP_L_ and HO-1 together, allows the vast majority (73%) of AML cells to become sensitive to TNF-induced death, compared to only 6% of the same cells dying in the absence of TNF. Interestingly, knockdown of HO-1 alone allowed only 15% of AML cells to be sensitive towards TNF-induced death. These findings suggest that together FLIP_L_ and HO-1 protect AML cells from TNF-induced apoptosis and both need to be targeted for effective AML therapy.

## DISCUSSION

Understanding host responses that modulate apoptosis in human AML is key to developing new therapeutic strategies to reduce the mortality associated with this complex disease. The present study demonstrates that FLIP_L_ can regulate the expression of HO-1, and that targeting knockdown of both molecules using siRNA improves apoptosis in response to TNF treatment. Most research to date regarding the importance of FLIP in regulating cell survival has focused on its role in preventing the formation of the DISC. This is the first time FLIP has been shown to regulate HO-1 gene induction in human cells, moreover, targeting both HO-1 and FLIP in combination could markedly improve survival rates in human AML.

The regulation of apoptosis in human AML is the focus of our research. We have previously shown that inhibiting HO-1 and NF-κB in combination allows AML cells to undergo apoptosis, thus suggesting that both these pathways are needed for the survival of these malignant cells [13]. HO-1 protects AML cells from apoptosis through its antioxidant metabolites bilirubin and carbon monoxide, which sequester free radicals associated with modern chemotherapies [15]. FLIP_L_ which is induced in AML in response to TNF in a NF-κB-dependent manner, is able to inhibit apoptosis by modulating activation of procaspase-8 and thereby inhibiting formation of the DISC [5]. We found in this study that by removing the induction of FLIP_L_ but not FLIP_S_ or FLIP_R_, induced the expression of the cytoprotective protein HO-1.

Expression of HO-1 is usually increased in human cancer cells, however we have recently shown that in human AML cells HO-1 expression is significantly lower when compared with nonmalignant control cells [13]. Further analysis found that the NF-κB subunits p50 and p65 negatively regulated the expression of this HO-1 mRNA by binding to a putative κB-binding site located in the HO-1 gene upstream of the antioxidant response element (ARE) which subsequently binds the transcription factor, Nrf2. Interestingly, FLIP has been shown to regulate NF-κB responses in human cells [14]. Thus, one explanation for the difference of HO-1 levels in cells with FLIP_L_-targeted knockdown compared to control cells, is that removing FLIP_L_ reduces the active NF-κB levels in human AML cells, thus removing its negative influence on the HO-1 promoter. This would allow HO-1 levels to come back to normal non-malignant control levels that are observed in ordinary hematopoietic stem cells (HSC).

We have previously shown that AML cells protect themselves from TNF-induced apoptotic responses by two interconnected mechanisms. The first is the induction of NF-κB-driven anti-apoptotic gene FLIP_L_. However when we remove NF-κB activation using BAY-11-7082, a secondary antiapoptotic pathway regulated by the transcription factor Nrf2, becomes apparent and drives the induction of cytoprotective gene HO-1. In this study we show that we do get some TNF-induced apoptosis of AML cells when we knockdown FLIP_L_ using siRNA, however when we combine the treatment to targeting HO-1 as well, we get a markedly increased level of apoptosis in response to TNF. This suggests that both FLIP_L_ and HO-1 are two genes that when individually targeted have little or no effect on apoptosis of AML cells, but targeting these two genes in combination would provide a novel mechanism that future more successful therapies must employ to effectively treat AML.

Our results clearly indicate that FLIP_L_ and HO-1 together have a role in controlling TNF-induced apoptotic responses in AML cells. It is also clear that knockdown of FLIP_L_ induces HO-1 expression in human AML, but not corresponding non-malignant control cells The role of the other FLIP isoforms, FLIP_S_ and FLIP_R_, is clearly less critical in AML cells in response to TNF. Moreover, the fact that we were unable to detect FLIP_R_ protein makes it even less of a target molecule for future anti-AML therapy. Previously we have shown that HO-1 may play an important secondary role in protecting AML cells from apoptotic responses, here we cement the concept that HO-1 is indeed very important for the survival of AML cells and therefore should be targeted with new SACT.

**Table 1:** AML sample information. This table defines the nature of the AML disease including WHO diagnosis and cytogenetics. Percent blast denotes the percentage of AML blasts after purification using density gradient centrifugation and in some instances CD34+ positive selection. Previous treatments are as outlined [16].

Number	Age	Gender	WHO diagnosis	Cytogenetics	% Blasts	Previous treatment
AML212	64	male	AML with t(8;21)(q22;q22);RUNX1-RUNX1T1	t(8;21)	85	nil
AML217	82	female	AML with myelodysplasia related changes	deletion 13	85	Hydroxycarbamide
AML301	46	female	AML with maturation	+4,+8, t(9;22)	70	nil
AML303	40	male	Acute promyelocytic leukaemia with t(15;17)(q22:q12) PML-RARA	t(15;17)	95	1999 DAT,DAT MACE,MiDAC (ref 16)
AML306	78	male	AML with myelodysplasia related changes	not available	85	nil

## MATERIALS AND METHODS

### Human research/patient rights

Primary AML cells were obtained under local ethical approval (LREC ref 07/H0310/146). Informed consent was obtained from patients and any identifying information was anonymised according to the approved ethics code of practice.

### Materials

The AML-derived cell lines THP-1 and HL60 were obtained from the European Collection of Cell Cultures. Anti-human HO-1 antibody was purchased from Assay Designs (Ann Arbor, MI). Anti-human FLIP antibody was purchased from Abcam (Cambridge, MA). Control, HO-1, FLIP_L_, FLIP_S_ and FLIP_R_ siRNA were purchased from Applied Biosystems (Foster City, CA). All other reagents were obtained from Sigma-Aldrich (St Louis, MO), unless indicated.

### Cell culture

For primary cell isolation, heparinized blood was collected from volunteers and human peripheral blood mononuclear cells (PBMCs) isolated by Histopaque (Sigma-Aldrich) density gradient centrifugation. PBMCs (4 x 10^6^/mL) were incubated in complete medium for 2 h at 37°C to allow adherence of monocytes. Positive selection of human hematopoietic stem cells (HSC) were isolated from PBMCs using a CD34 positive selection kit (Miltenyi Biotec, Auburn, CA). For all CD34+ and primary monocyte experiments at least three different donors were used to obtain the results presented in this paper. AML samples that were less than 80% blasts and expressed CD34, were purified using the CD34 positive selection kit. Cell type was confirmed by microscopy and flow cytometry.

### RNA extraction and real-time PCR

Total RNA was extracted from 5 x 10^5^ cells using the Nucleic acid PrepStation from Applied Biosystems, according to the manufacturer's instructions. Reverse transcription was performed using the RNA polymerase chain reaction (PCR) core kit (Applied Biosystems). Real-time PCR primers were purchased from Invitrogen. Relative quantitative real-time PCR used SYBR green technology (Roche) on cDNA generated from the reverse transcription of purified RNA. After preamplification (95°C for 2 min), the PCRs were amplified for 45 cycles (95°C for 15 s and 60°C for 10 s and 72°C for 10 s) on a LightCycler 480 (Roche). mRNA expression was normalized against GAPDH expression using the standard curve method.

### Western immunoblotting, binding assay and flow cytometry

SDS-PAGE and Western analyses were performed as described previously [12]. Briefly, whole cell lysates were extracted using radioimmunoprecipitation assay (RIPA) buffer method. Western blot protein detection was by enhanced chemiluminescence (ECL). Flow cytometry for measuring apoptosis was performed on an Accuri C6 flow cytometer (Acurri).

### Statistical analyses

Student's *t* test was performed to assess statistical significance from controls. Results with P < 0.05 were considered statistically significant (*). Results represent the mean ± SEM of 3 independent experiments. For Western blotting experiments, data are representative of at least 3 separate experiments.
